# Efficacy and safety of canagliflozin in patients with type 2 diabetes based on history of cardiovascular disease or cardiovascular risk factors: a post hoc analysis of pooled data

**DOI:** 10.1186/s12933-017-0517-7

**Published:** 2017-03-21

**Authors:** Michael J. Davies, Katherine Merton, Ujjwala Vijapurkar, Jacqueline Yee, Rong Qiu

**Affiliations:** 1 Janssen Scientific Affairs, LLC, 1125 Trenton-Harbourton Road, Titusville, NJ 08560 USA; 2 Janssen Research & Development, LLC, 920 US Highway 202 South, Raritan, NJ 08869 USA

**Keywords:** Canagliflozin, Type 2 diabetes mellitus, Cardiovascular disease, SGLT2 inhibitor, Risk factors

## Abstract

**Background:**

Treatment of patients with type 2 diabetes mellitus (T2DM) and a history of cardiovascular (CV) disease or CV risk factors may present clinical challenges due to the presence of comorbid conditions and the use of concomitant medications. The sodium glucose co-transporter 2 inhibitor, canagliflozin, has been shown to improve glycaemic control and reduce body weight and blood pressure (BP) with a favourable tolerability profile in a broad range of patients with T2DM. This post hoc analysis assessed the efficacy and safety of canagliflozin in patients with T2DM based on CV disease history or CV risk factors.

**Methods:**

Analyses were based on pooled data from four 26-week, placebo-controlled, Phase 3 studies that evaluated canagliflozin 100 and 300 mg in patients with T2DM (N = 2313; mean HbA1c, 8.0%; body weight, 89 kg; systolic BP, 128 mmHg). Changes from baseline to week 26 in HbA1c, body weight, and systolic BP were assessed based on history of CV disease, history of hypertension, baseline statin use, and number of CV risk factors. Safety was assessed based on adverse event (AE) reports.

**Results:**

At week 26, both canagliflozin doses lowered HbA1c, body weight, and systolic BP compared with placebo in patients with and without CV disease history or risk factors. Placebo-subtracted HbA1c reductions with canagliflozin 100 and 300 mg were similar in patients with a history of CV disease (−0.95 and −1.07%) versus no history of CV disease (−0.71 and −0.90%), history of hypertension (−0.72 and −0.89%) versus no history of hypertension (−0.73 and −0.95%), baseline statin use (−0.77 and −0.99%) versus no statin use (−0.69 and −0.85%), and 0–1 CV risk factor (−0.72 and −0.87%) versus ≥2 CV risk factors (−0.74 and −1.02%). Similar body weight and systolic BP reductions were seen with canagliflozin versus placebo across subgroups. The incidence of AEs, AEs leading to discontinuation, and serious AEs was similar across subgroups.

**Conclusions:**

The efficacy and safety of canagliflozin were generally consistent across subgroups of patients with T2DM and varying degrees of CV disease history or risk factors.

*Trial registration numbers and dates* ClinicalTrials.gov: NCT01081834, 4 March 2010; NCT01106625, 1 April 2010; NCT01106677, 1 April 2010; NCT01106690, 1 April 2010

**Electronic supplementary material:**

The online version of this article (doi:10.1186/s12933-017-0517-7) contains supplementary material, which is available to authorized users.

## Background

Type 2 diabetes mellitus (T2DM) affects roughly 400 million adults across the globe and contributes to 5 million deaths annually [1]. The majority of these deaths are a result of cardiovascular (CV) complications, which are very common in patients with T2DM due to poorly controlled chronic hyperglycaemia and reduced insulin sensitivity [2–4]. Other major contributors to increased CV risk in patients with T2DM include comorbid conditions such as hypertension and dyslipidaemia [5, 6]. The presence of these comorbid conditions and the associated requirements for concomitant medication use can present significant clinical challenges in the treatment of patients with T2DM and a history of CV disease or CV risk factors [7, 8].

Some pharmacologic agents may not be suitable for patients with T2DM and existing CV disease or CV risk factors. For instance, the prescribing information for the sulphonylureas glipizide [9] and glyburide [10] include a warning for increased risk of CV death, and the prescribing information for pioglitazone includes a warning for congestive heart failure [11]. Additionally, guidelines from the American Diabetes Association recommend against the use of agents associated with hypoglycaemia in patients with T2DM and coronary artery disease [7]. Overall, there remains a need for antihyperglycaemic agents (AHAs) that are efficacious and well tolerated in people with T2DM and CV disease history/risk factors; ideally, such medications would provide not only improved glycaemic control but also favourable effects on CV risk factors such as body weight, hypertension, and dyslipidaemia.

Canagliflozin is a sodium glucose co-transporter 2 (SGLT2) inhibitor approved for the treatment of adults with T2DM. Canagliflozin lowers the renal threshold for glucose, thereby promoting urinary glucose excretion (UGE) and resulting in a mild osmotic diuresis and a net caloric loss [12, 13]. The mechanism of action for canagliflozin is independent of insulin and is complementary to other AHAs, with a low inherent risk for hypoglycaemia. Across Phase 3 clinical trials, canagliflozin has been shown to provide improvements in glycaemic control as well as reductions in body weight and blood pressure (BP) as monotherapy and in combination with other AHAs in a broad range of patients with T2DM [14].

In this analysis, the efficacy and safety of canagliflozin was assessed among patients with T2DM in subgroups based on CV disease history and CV risk factors using pooled data from four 26-week, placebo-controlled, Phase 3 studies [15–18].

## Methods

### Patients and study design

These post hoc analyses were based on pooled data from four 26-week, randomised, placebo-controlled, Phase 3 studies of canagliflozin 100 and 300 mg in patients with T2DM. These studies included evaluation of canagliflozin compared with placebo as monotherapy in patients with T2DM inadequately controlled with diet and exercise (ClinicalTrials.gov Identifier: NCT01081834) [15], and as combination therapy in patients on background metformin (NCT01106677) [16], metformin plus sulphonylurea (NCT01106625) [17], and metformin plus pioglitazone (NCT01106690) [18]. In all studies, patients were randomised to receive canagliflozin 100 or 300 mg or placebo once daily during a 26-week, double-blind, core treatment period, followed by a 26-week extension period. Data from the 26-week core treatment periods of each study were included in this pooled analysis. The high glycaemic subset (HbA1c >10 and ≤12.0%) of the monotherapy study [15] was not placebo controlled, and the sitagliptin arm of the add-on to metformin study [16] was not prespecified for efficacy comparisons versus canagliflozin at week 26; therefore, these populations were excluded from the analysis.

Key inclusion criteria for these studies are summarised in Table 1. Key exclusion criteria common to all studies included repeated fasting plasma glucose (FPG) generally ≥15.0 mmol/L during the pretreatment phase; history of diabetic ketoacidosis or type 1 diabetes; history of myocardial infarction, unstable angina, revascularisation procedure, or cerebrovascular accident within 3 months of screening; uncontrolled hypertension; and alanine aminotransferase level >2 times the upper limit or normal or total bilirubin >1.5 times the upper limit of normal at screening [19].

**Table 1:** Study design and patient population

Study	Inclusion criteria			Patients contributing data to pooled analysis, n			
	Age, years	HbA1c, %	eGFR, mL/min/1.73 m^2^	PBO	CANA 100 mg	CANA 300 mg	Total
Monotherapy	18–80	7.0–10.0	≥50	192	195	197	584
Add-on to MET	18–80	7.0–10.5	≥55	183	368	367	918
Add-on to MET + SU	18–80	7.0–10.5	≥55	156	157	156	469
Add-on to MET + PIO	18–80	7.0–10.5	≥55	115	113	114	342
Overall total, N				646	833	834	2313

Data have been previously reported [19, 22, 24]

*eGFR* estimated glomerular filtration rate, *PBO* placebo, *CANA* canagliflozin, *MET* metformin, *SU* sulphonylurea, *PIO* pioglitazone

Details of the individual study designs have been previously reported [15–18]. Briefly, in each study, eligible patients who were on protocol-specified background AHA treatment entered into a 2-week, placebo run-in period. Patients who were not on protocol-specified background AHA treatment (n = 821; 35.5%) entered an 8- to 12-week adjustment/dose stabilisation period prior to the placebo run-in period. After the placebo run-in period, patients were randomised (1:1:1) to canagliflozin 100 or 300 mg or placebo. Glycaemic rescue therapy using an AHA that was complementary to the protocol-specified background therapy was initiated using protocol-specified FPG criteria.

All studies were conducted in accordance with ethical principles originating in the Declaration of Helsinki and were consistent with Good Clinical Practices and applicable regulatory requirements. Approval was obtained from institutional review boards and independent ethics committees for participating centres, and written informed consent was provided by all patients prior to participation.

### Study endpoints and assessments

For this post hoc analysis, data from patients who received canagliflozin 100 or 300 mg or placebo in these four clinical trials were pooled and analysed in four different subgroups: (1) history of CV disease (yes/no); (2) history of hypertension (yes/no); (3) statin use at baseline (yes/no); and (4) number of CV risk factors at baseline (0–1 or ≥2), defined as current cigarette smoker, T2DM history of ≥10 years, baseline high-density lipoprotein cholesterol (HDL-C) of <39 mg/dL, micro- or macro-albuminuria (i.e., baseline albumin to creatinine ratio of ≥30 mg/g), and screening systolic BP >140 mmHg. The terms used to define history of CV disease or history of hypertension and statin use at baseline are provided in Additional file 1. Efficacy endpoints assessed at week 26 for each subgroup included changes from baseline in HbA1c, body weight, and systolic BP. Safety assessments across subgroups included overall incidence of adverse events (AEs), AEs leading to discontinuation, AEs related to study drug, serious AEs, and deaths.

### Statistical analyses

All analyses used data from the modified intent-to-treat (mITT) population from each study, which consisted of all randomised patients who received ≥1 dose of double-blind study drug. Missing data at week 26 were imputed using the last observation carried forward (LOCF). For patients who received glycaemic rescue therapy, the last post-baseline value prior to the initiation of rescue therapy was used for efficacy analyses. Efficacy endpoints were assessed using an analysis of covariance (ANCOVA) model, with treatment and study factors and the representative baseline value as a covariate. Least squares (LS) means and 95% confidence intervals (CIs) were estimated for comparisons of each canagliflozin dose with placebo. Statistical testing of canagliflozin versus placebo was not prespecified for analyses of efficacy parameters in these post hoc analyses. Therefore, no *P* values are reported; however, 95% CIs are provided for descriptive purposes.

## Results

### Patients

**Fig. 1:**
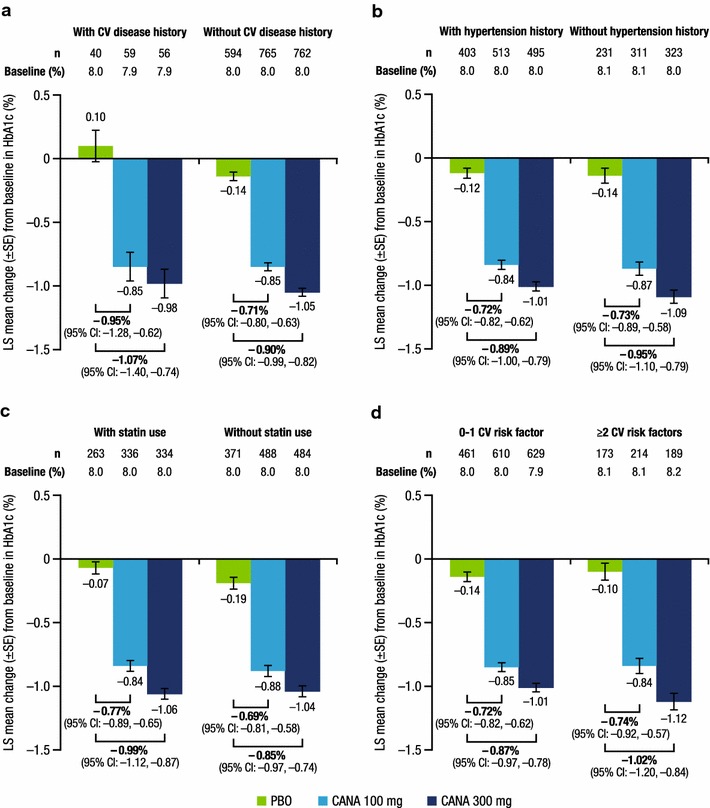
Change from baseline in HbA1c at week 26. **a** History of CV disease, **b** history of hypertension, **c** baseline statin use, **d** number of CV risk factors. *CV* cardiovascular, *LS* least squares, *SE* standard error, *CI* confidence interval, *PBO* placebo, *CANA* canagliflozin

**Fig. 2:**
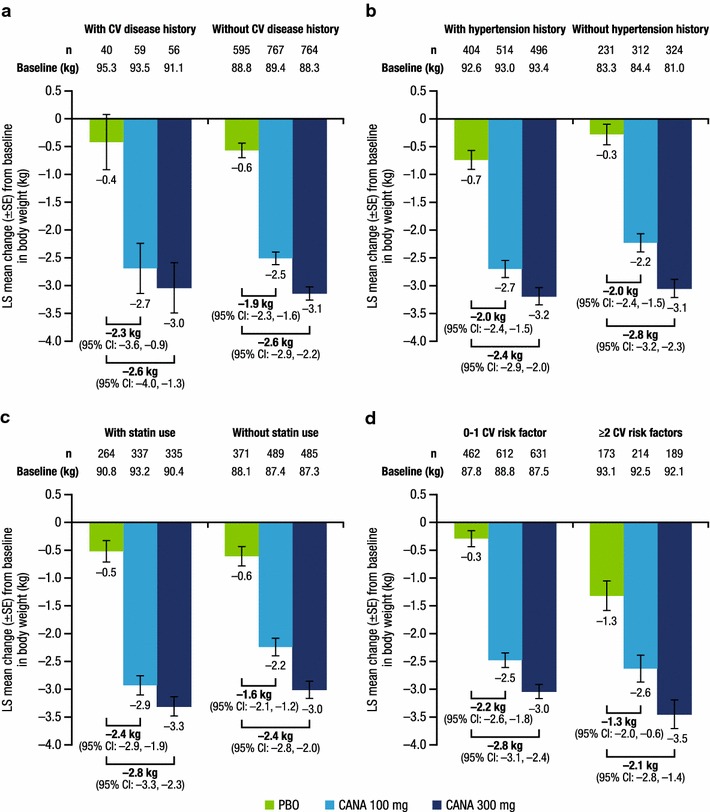
Change from baseline in body weight at week 26. **a** History of CV disease, **b** history of hypertension, **c** baseline statin use, **d** number of CV risk factors. *CV* cardiovascular, *LS* least squares, *SE* standard error, *CI* confidence interval, *PBO* placebo, *CANA* canagliflozin

**Fig. 3:**
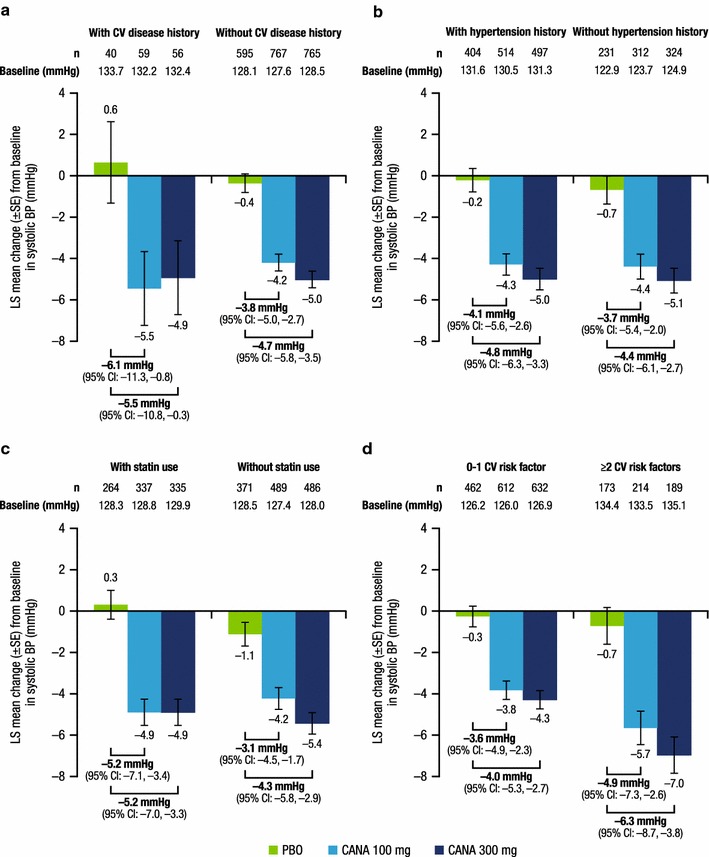
Change from baseline in systolic BP at week 26. **a** History of CV disease, **b** history of hypertension, **c** baseline statin use, **d** number of CV risk factors. *BP* blood pressure, *CV* cardiovascular, *LS* least squares, *SE* standard error, *CI* confidence interval, *PBO* placebo, CANA canagliflozin

A total of 2313 patients were included in the mITT population; of these, 155 patients (6.7%) had a history of CV disease, 1433 (62.0%) had a history of hypertension, and 945 (40.9%) were using statins at baseline; 1727 patients (74.7%) had 0 or 1 CV risk factor and 586 (25.3%) had ≥2 CV risk factors. In the overall population, patient demographic and disease characteristics were generally balanced across treatment groups (Table 2). Baseline HbA1c, body weight, and systolic BP values in subgroups by CV disease history or CV risk factors are shown in Figs. 1, 2, 3. Generally, patients in the higher CV risk subgroups had higher baseline body weight and systolic BP values.

**Table 2:** Baseline demographic and disease characteristics (overall population)

Characteristic^a^	PBO (n = 646)	CANA 100 mg (n = 833)	CANA 300 mg (n = 834)
Sex, n (%)			
Male	334 (52)	408 (49)	404 (48)
Female	312 (48)	425 (51)	430 (52)
Age, years	56.3 (9.8)	55.9 (10.1)	55.7 (9.5)
Race, n (%)			
White	470 (73)	591 (71)	610 (73)
Black or African American	28 (4)	43 (5)	48 (6)
Asian	82 (13)	103 (12)	100 (12)
Other^b^	66 (10)	96 (12)	76 (9)
HbA1c, %	8.0 (0.9)	8.0 (0.9)	8.0 (1.0)
Body weight, kg	89.3 (21.7)	89.8 (22.3)	88.5 (22.0)
Systolic BP, mmHg	128.2 (13.3)	128.0 (12.8)	128.8 (12.8)
eGFR, mL/min/1.73 m^2^	87.0 (19.8)	88.3 (19.0)	88.8 (18.9)
Duration of T2DM, years	7.5 (6.2)	7.2 (5.8)	7.4 (6.2)

Data have been previously reported [19, 22, 24]

*PBO* placebo, *CANA* canagliflozin, *BP* blood pressure, *eGFR* estimated glomerular filtration rate, *T2DM* type 2 diabetes mellitus, *SD* standard deviation

^a^Data are mean (SD) unless otherwise indicated

^b^Includes American Indian or Alaska Native, Native Hawaiian or other Pacific Islander, multiple, other, unknown, and not reported

### Efficacy

As shown in Fig. 1, LS mean changes in HbA1c from baseline to week 26 were greater with canagliflozin 100 and 300 mg than with placebo in all subgroups. Placebo-subtracted reductions in HbA1c were similar across subgroups, regardless of the presence or absence of history of CV disease or history of hypertension, baseline statin use, or 0–1 versus ≥2 CV risk factors. LS mean changes in body weight (Fig. 2) and systolic BP (Fig. 3) from baseline to week 26 were also greater with canagliflozin 100 and 300 mg than with placebo in all subgroups. Placebo-subtracted reductions in body weight were similar across subgroups, regardless of the presence or absence of history of CV disease or history of hypertension, baseline statin use, or 0–1 versus ≥2 CV risk factors.

### Safety

Canagliflozin 100 and 300 mg were generally well tolerated across subgroups by CV disease history or CV risk factors. The incidence of overall AEs, AEs leading to discontinuation, and serious AEs was similar with canagliflozin 100 and 300 mg and placebo across subgroups (Table 3).

**Table 3:** Overall safety summary

Patients, n (%)	History of CV disease						History of hypertension					
	Yes			No			Yes			No		
	PBO (n = 40)	CANA 100 mg (n = 59)	CANA 300 mg (n = 56)	PBO (n = 606)	CANA 100 mg (n = 774)	CANA 300 mg (n = 778)	PBO (n = 409)	CANA 100 mg (n = 518)	CANA 300 mg (n = 506)	PBO (n = 237)	CANA 100 mg (n = 315)	CANA 300 mg (n = 328)
Any AE	27 (67.5)	32 (54.2)	42 (75.0)	357 (58.9)	469 (60.6)	452 (58.1)	253 (61.9)	300 (57.9)	298 (58.9)	131 (55.3)	201 (63.8)	196 (59.8)
AEs leading to discontinuation	0	4 (6.8)	3 (5.4)	20 (3.3)	32 (4.1)	27 (3.5)	13 (3.2)	25 (4.8)	23 (4.5)	7 (3.0)	11 (3.5)	7 (2.1)
AEs related to study drug^a^	5 (12.5)	8 (13.6)	18 (32.1)	80 (13.2)	163 (21.1)	173 (22.2)	62 (15.2)	101 (19.5)	111 (21.9)	23 (9.7)	70 (22.2)	80 (24.4)
Serious AEs	2 (5.0)	2 (3.4)	4 (7.1)	20 (3.3)	26 (3.4)	18 (2.3)	19 (4.6)	17 (3.3)	11 (2.2)	3 (1.3)	11 (3.5)	11 (3.4)
Deaths	0	0	1 (1.8)	2 (0.3)	1 (0.1)	0	2 (0.5)	1 (0.2)	1 (0.2)	0	0	0

Patients, n (%)	Baseline statin use						CV risk factors					
	Yes			No			0–1			≥2		
	PBO (n = 266)	CANA 100 mg (n = 339)	CANA 300 mg (n = 340)	PBO (n = 380)	CANA 100 mg (n = 494)	CANA 300 mg (n = 494)	PBO (n = 468)	CANA 100 mg (n = 616)	CANA 300 mg (n = 643)	PBO (n = 178)	CANA 100 mg (n = 217)	CANA 300 mg (n = 191)
Any AE	168 (63.2)	212 (62.5)	220 (64.7)	216 (56.8)	289 (58.5)	274 (55.5)	275 (58.8)	380 (61.7)	381 (59.3)	109 (61.2)	121 (55.8)	113 (59.2)
AEs leading to discontinuation	8 (3.0)	17 (5.0)	17 (5.0)	12 (3.2)	19 (3.8)	13 (2.6)	14 (3.0)	23 (3.7)	24 (3.7)	6 (3.4)	13 (6.0)	6 (3.1)
AEs related to study drug^a^	42 (15.8)	75 (22.1)	95 (27.9)	43 (11.3)	96 (19.4)	96 (19.4)	61 (13.0)	130 (21.1)	148 (23.0)	24 (13.5)	41 (18.9)	43 (22.5)
Serious AEs	12 (4.5)	13 (3.8)	12 (3.5)	10 (2.6)	15 (3.0)	10 (2.0)	13 (2.8)	18 (2.9)	17 (2.6)	9 (5.1)	10 (4.6)	5 (2.6)
Deaths	1 (0.4)	0	0	1 (0.3)	1 (0.2)	1 (0.2)	2 (0.4)	0	1 (0.2)	0	1 (0.5)	0

*CV* cardiovascular, *PBO* placebo, *CANA* canagliflozin, *AE* adverse event

^a^Possibly, probably, or very likely related to study drug, as assessed by investigators

## Discussion

Patients with T2DM are at increased risk for CV disease, in part because of increased prevalence of comorbidities such as hypertension and dyslipidaemia [20, 21]. The presence of CV disease and CV risk factors can complicate T2DM management [8]. This post hoc analysis evaluated the efficacy and safety of canagliflozin 100 and 300 mg in patients with T2DM based on history of CV disease, history of hypertension, baseline use of statins, and the number of CV risk factors. To have a sufficient sample size for analysis that was representative of a diverse population of patients with T2DM, data were pooled from four similarly designed 26-week, placebo-controlled studies of canagliflozin. Results from these analyses showed that treatment with canagliflozin provided meaningful reductions in HbA1c, body weight, and systolic BP that were similar regardless of the presence or absence of history of CV disease, history of hypertension, baseline statin use, or number of CV risk factors. Across subgroups by CV disease history or risk factors, both doses of canagliflozin were generally well tolerated, with a safety profile consistent with that reported across clinical trials of canagliflozin [14, 22, 23]. In the overall population, the incidence of AEs was similar with canagliflozin and placebo; not surprisingly, an increased incidence of AEs related to the mechanism of SGLT2 inhibition (e.g., genital mycotic infections, osmotic diuresis–related AEs) was seen with canagliflozin [19, 22, 24].

Given the high burden of CV disease among patients with T2DM, some guidelines recommend a multifactorial approach to managing T2DM [7, 25]. SGLT2 inhibitors, such as canagliflozin, have been shown to provide significant improvements in HbA1c, body weight, and BP in a broad range of patients with T2DM [23], including older patient populations (≥65 and ≥75 years of age) who may be at elevated risk of CV disease [26, 27]. To improve dyslipidaemia, the European Society of Cardiology recommends statin therapy for all patients with T2DM over 40 years of age and for selected younger patients with elevated CV disease risk [28]. Despite this recommendation, less than 50% of patients in this population were using statins at baseline. Statin therapy has been shown to be associated with increased HbA1c levels in patients with T2DM [29–32]. In the current analysis, canagliflozin treatment provided clinically meaningful improvements in HbA1c in patients with T2DM, regardless of baseline statin use.

Choice of AHAs may be limited for patients with T2DM and CV disease history and CV risk factors based on data suggesting an increased risk for negative CV outcomes with some agents [9–11]. There is a growing body of evidence that SGLT2 inhibitors can provide cardiometabolic benefits beyond glycaemic control. In the EMPA-REG OUTCOME trial, empagliflozin showed a significant reduction in the risk of CV death and heart failure hospitalisation compared with placebo [33]. Based on these results, the US Food and Drug Administration recently approved a new indication for empagliflozin to reduce the risk of CV death in adult patients with T2DM and established CV disease [34]. It is hypothesised that the mechanism for the cardioprotective effect seen with empagliflozin is likely relevant for the entire SGLT2 inhibitor class due to improvements in glycaemic control, as well as BP and body weight reduction via induction of a mild osmotic diuresis, increased natriuresis, and net caloric loss [35–38]. In particular, it has been postulated that the increased osmotic diuresis associated with SGLT2 inhibition may help to reduce cardiac workload via reductions in BP and intravascular volume [37, 38]. Results from the ongoing CANVAS Program [39], including CANagliflozin cardioVascular Assessment Study (CANVAS; ClinicalTrials.gov Identifier: NCT01032629) and CANVAS-R (renal endpoints; NCT01989754) [40, 41], will provide evidence on the CV safety and efficacy of canagliflozin in more than 10,000 patients with CV disease history or CV risk factors upon completion in 2017 and will confirm whether the CV benefits observed with empagliflozin support a class effect. In addition, a separate study is underway to evaluate the effects of canagliflozin versus glimepiride in Japanese patients with T2DM and chronic heart failure [42].

Due to the post hoc nature of these analyses, the current study was limited by a lack of prespecified statistical testing across subgroups. However, calculated 95% CIs allowed for descriptive comparisons for treatment with both canagliflozin doses and placebo. Comparisons were also limited by the small number of patients with CV disease history at baseline, which was not surprising for this general population of patients with T2DM. Nevertheless, the subgroup analysis results were generally consistent with those seen in the overall pooled population [24]. Similar analyses using longer-term efficacy and safety data could provide additional insight into the durability of benefits and risks associated with canagliflozin based on CV disease history and CV risk factors.

## Conclusions

Treatment with canagliflozin 100 and 300 mg provided consistent reductions in HbA1c, body weight, and systolic BP, and was generally well tolerated over 26 weeks of treatment in patients with T2DM, regardless of CV disease history or CV risk factors. These findings are noteworthy given the high burden of CV disease among patients with T2DM and the need for diabetes therapies that are safe and efficacious in patients with T2DM and CV disease.

## Additional file

**Additional file 1.** Terms used to define history of CV disease, history of hypertension, and statin use at baseline.

## Abbreviations

AE: adverse event
AHA: antihyperglycaemic agent
ANCOVA: analysis of covariance
ATC: Anatomical Therapeutic Chemical
BMI: body mass index
BP: blood pressure
CANA: canagliflozin
CANVAS: CANagliflozin cardioVascular Assessment Study
CI: confidence interval
CV: cardiovascular
eGFR: estimated glomerular filtration rate
FPG: fasting plasma glucose
HDL-C: high-density lipoprotein cholesterol
LOCF: last observation carried forward
LS: least squares
MET: metformin
mITT: modified intent-to-treat
PBO: placebo
PIO: pioglitazone
SD: standard deviation
SE: standard error
SGLT2: sodium glucose co-transporter 2
SU: sulphonylurea
T2DM: type 2 diabetes mellitus
UGE: urinary glucose excretion
