# Supplementary material for: Efficacy and safety of canagliflozin in patients with type 2 diabetes based on history of cardiovascular disease or cardiovascular risk factors: a post hoc analysis of pooled data
Source: Cardiovasc Diabetol. 2017 Mar 21;16:40. doi: 10.1186/s12933-017-0517-7 (PMC5361783; doi:10.1186/s12933-017-0517-7)
Supplement: Supplementary file 1 — Additional file 1. Terms used to define history of CV disease, history of hypertension, and statin use at baseline. [file 12933_2017_517_MOESM1_ESM.docx]

**Additional File 1. Terms Used to Define History of CV Disease, History of Hypertension, and Statin Use at Baseline**

1. **Terms Used to Define History of CV Disease**

| - Basal ganglia haemorrhage | - Brain stem infarction |
| --- | --- |
| - Brain stem haemorrhage | - Brain stem thrombosis |
| - Brain stem stroke | - Carotid artery thrombosis |
| - Cerebellar haematoma | - Cerebellar artery thrombosis |
| - Cerebellar haemorrhage | - Cerebellar embolism |
| - Cerebral arteriovenous malformation haemorrhagic | - Cerebellar infarction |
| - Cerebral haematoma | - Cerebral artery embolism |
| - Cerebral haemorrhage | - Cerebral artery thrombosis |
| - Cerebral microhaemorrhage | - Cerebral infarction |
| - Cerebrovascular accident | - Cerebral thrombosis |
| - Cerebrovascular disorder | - Embolic cerebral infarction |
| - Haemorrhagic cerebral infarction | - Embolic stroke |
| - Haemorrhagic stroke | - Ischaemic cerebral infarction |
| - Haemorrhagic transformation stroke | - Ischaemic stroke |
| - Intraventricular haemorrhage | - Lacunar infarction |
| - Putamen haemorrhage | - Post procedural stroke |
| - Stroke in evolution | - Thalamic infarction |
| - Subarachnoid haemorrhage | - Thrombotic cerebral infarction |
| - Thalamus haemorrhage | - Thrombotic stroke |
| - Basal ganglia infarction | - Vertebral artery thrombosis |
| - Basilar artery thrombosis | - Acute coronary syndrome |
| - Acute myocardial infarction | - Coronary revascularisation |
| - Coronary artery embolism | - In-stent coronary artery restenosis |
| - Coronary artery occlusion | - Microvascular angina |
| - Coronary artery reocclusion | - Percutaneous coronary intervention |
| - Coronary artery thrombosis | - Arteriogram coronary abnormal |
| - Coronary bypass thrombosis | - Cardiac stress test abnormal |
| - Myocardial infarction | - Exercise test abnormal |
| - Myocardial reperfusion injury |  |
| - Papillary muscle infarction |  |
| - Post procedural myocardial infarction |  |
| - Postinfarction angina |  |
| - Scan myocardial perfusion abnormal |  |
| - Angina unstable |  |
| - Coronary angioplasty |  |
| - Coronary arterial stent insertion |  |
| - Coronary artery bypass |  |
| - Coronary artery disease |  |
| - Coronary artery insufficiency |  |
| - Coronary artery restenosis |  |
| - Coronary artery stenosis |  |
| - Coronary endarterectomy |  |
| - Coronary no-reflow phenomenon |  |
| - Coronary ostial stenosis |  |

1. **Terms Used to Define History of Hypertension**

- Essential hypertension
- Hypertension
- Hypertensive angiopathy
- Hypertensive cardiomyopathy
- Hypertensive crisis
- Hypertensive heart disease
- Retinopathy hypertensive

1. **Terms Used to Define Statin Use at Baseline**

- Anatomical Therapeutic Chemical (ATC) codes for HMG-CoA reductase inhibitors (C10AA, C10BA, and C10BX)
